# ^177^Lu-DOTA-HYNIC-Lys(Nal)-Urea-Glu: Biokinetics, Dosimetry, and Evaluation in Patients with Advanced Prostate Cancer

**DOI:** 10.1155/2018/5247153

**Published:** 2018-11-11

**Authors:** Clara Santos-Cuevas, Guillermina Ferro-Flores, Francisco O. García-Pérez, Nallely Jiménez-Mancilla, Gerardo Ramírez-Nava, Blanca Ocampo-García, Myrna Luna-Gutiérrez, Erika Azorín-Vega, Jenny Davanzo, Irma Soldevilla-Gallardo

**Affiliations:** ^1^Departamento de Materiales Radiactivos, Instituto Nacional de Investigaciones Nucleares (ININ), Ocoyoacac 52750, Estado de México, Mexico; ^2^Departamento de Medicina Nuclear, Instituto Nacional de Cancerología, Ciudad de México 14000, Mexico; ^3^CONACyT, Instituto Nacional de Investigaciones Nucleares (ININ), Ocoyoacac 52750, Estado de México, Mexico; ^4^Departamento de Posgrado, Instituto Politécnico Nacional, Ciudad de México 07340, Mexico; ^5^Unidad de de Medicina Nuclear, Centro Médico ABC Campus Observatorio, Ciudad de México 01120, Mexico

## Abstract

SPECT/CT images in patients have demonstrated the ability of [^99m^Tc]Tc-EDDA/HYNIC-Lys(Nal)-Urea-Glu ([^99m^Tc]Tc-iPSMA) to detect tumors and metastases of prostate cancer. Considering that theranostics combines the potential of therapeutic and diagnostic radionuclides in the same molecular probe, the aim of this research was to estimate the biokinetics and dosimetry of ^177^Lu-DOTA-HYNIC-Lys(Nal)-Urea-Glu (^177^Lu-iPSMA) in healthy subjects and analyze the response in patients receiving ^177^Lu-iPSMA therapeutic doses. ^177^Lu-iPSMA was obtained from lyophilized formulations with radiochemical purities >98%. Whole-body images from five healthy subjects were acquired at 20 min, 6, 24, 48, and 120 h after ^177^Lu-iPSMA administration (185 MBq). The image sequence was used to extrapolate the ^177^Lu-iPSMA time-activity curves of each organ to adjust the biokinetic model and calculate the total number of disintegrations (*N*) that occurred in the source regions. *N* data were the input for the OLINDA/EXM code to calculate internal radiation doses. Ten patients (median age: 68 y; range 58–86 y) received from 1 to 4 cycles of ^177^Lu-iPSMA (3.7 or 7.4 GBq) every 8–10 weeks. Response was evaluated using the ^68^Ga-PSMA-ligand-PET/CT or ^99m^Tc-iPSMA-SPECT/CT diagnostic images and serum PSA levels before and after ^177^Lu-iPSMA treatment. The blood activity showed a half-life value of 1.1 h for the fast component (*T*_1/2_*α* = ln2/0.614), 9.2 h for the first slow component (*T*_1/2_*β* = ln2/0.075), and 79.6 h for the second slow component (*T*_1/2_*γ* = ln2/0.008). The average absorbed doses were 0.23, 0.28, 0.88, and 1.17 Gy/GBq for the spleen, liver, kidney, and salivary glands. A total of 18 cycles were performed in 10 patients. A PSA decrease and some reduction of the radiotracer uptake (SUV) in tumor lesions occurred in 60% and 70% of the patients, respectively. ^177^Lu-iPSMA obtained from kit formulations showed high tumor uptake with good response rates in patients. The results obtained in this study warrant further clinical studies to establish the optimal number of treatment cycles and for evaluating the effect of this therapeutic agent on survival of patients.

## 1. Introduction

Prostate-specific membrane antigen (PSMA) is a metallopeptidase overexpressed predominantly in prostate cancer (PCa) cells [1]. The therapeutic application of two different lutetium-177-labeled PSMA inhibitors ([^177^Lu]Lu-PSMA-617 and [^177^Lu]Lu-PSMA-I&T) has shown a decrease of >50% in the prostate antigen (PSA) levels and a significant survival increase in 70% of patients with metastatic PCa [2–4]. However, before any radiotherapeutic treatment, the radiopharmaceutical uptake in tumors must be evaluated by nuclear imaging to confirm whether the treatment will be useful for the patient.

Because of their high sensitivity and specificity, several ^68^Ga-PSMA inhibitors for PET/CT imaging of prostate cancer are currently used in clinical trials [5–7]. However, technetium-99m is still the most widely used radionuclide for diagnostic imaging. Recently, our group reported the preparation and biokinetics and dosimetry of [^99m^Tc]Tc-EDDA/HYNIC-iPSMA ([^99m^Tc]Tc-ethylenediamine-*N*,*N*′-diacetic acid (EDDA)/hydrazinonicotinyl(HYNIC)-Lys(Nal)-Urea-Glu) as a radiopharmaceutical with the ability to specifically detect PSMA expression in tumors of prostate cancer by SPECT/CT imaging [8–10].

**Figure 1:**
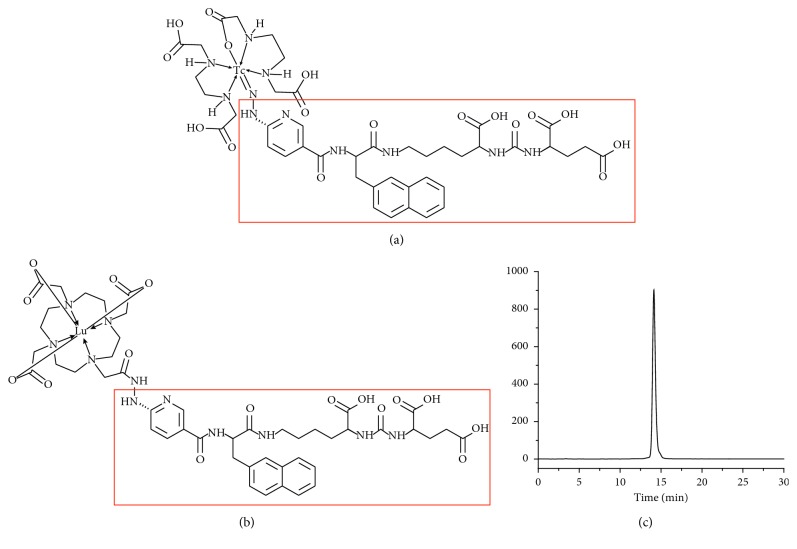
Schematic structure of the theranostic pair: (a) [^99m^Tc]Tc-EDDA/HYNIC-iPSMA ([^99m^Tc]Tc-iPSMA) and (b) ^177^Lu-DOTA-HYNIC iPSMA (^177^Lu-iPSMA). (c) Radio-HPLC analysis of ^177^Lu-iPSMA obtained from a multidose lyophilized kit before injection to patients with high radiochemical purity (>99%).

Considering that theranostics combines the potential of therapeutic and diagnostic radionuclides in the same molecular probe, we have also reported the synthesis, preparation, and preclinical studies of the therapeutic radiopharmaceutical ^177^Lu-DOTA-HYNIC-iPSMA [^177^Lu-(1,4,7,10-tetraazacyclododecane-*N*,*N*′,*N*″,*N*‴-tetraacetic acid)-HYNIC-Lys(Nal)-Urea-Glu] in order to develop a new theranostic ^99m^Tc/^177^Lu pair, useful in prostate cancer (Figure 1) [11, 12].

The aim of this study was to estimate the biokinetics and dosimetry of ^177^Lu-DOTA-HYNIC-iPSMA (^177^Lu-iPSMA) in five healthy subjects and analyze the response in ten patients with histologically confirmed prostate cancer that received therapeutic doses of ^177^Lu-iPSMA.

## 2. Materials and Methods

### 2.1. Reagents

The DOTA-HYNIC-iPSMA peptide conjugate (1,4,7,10-tetraazacyclododecane-*N*,*N*′,*N*″,*N*‴-tetraacetic acid-hydrazinonicotinyl-Lys(Nal)-Urea-Glu derivative, MW 1038 g/mol) was designed at ININ, and the synthesis was requested from Ontores Biotechnology Co., Ltd (Zhejiang, China), with a purity > 98% as analyzed by reversed-phase HPLC (RP-HPLC) and mass spectroscopy. Lutetium (^177^Lu) chloride was obtained from ITG, Germany (EndolucinBeta 40 GBq/mL, in sterile 0.04 M HCl solution, noncarrier-added). All the other reagents were purchased from Sigma-Aldrich Chemical Co. and were used as received.

### 2.2. Preparation of ^177^Lu-iPSMA

Lutetium-177-labeled iPSMA (^177^Lu-iPSMA) was prepared from a multidose lyophilized formulation under aseptic conditions in a GMP-certified facility according to the method described by Luna-Gutierrez et al. [11]. For the radiochemical synthesis, the ^177^LuCl_3_ vial (40 GBq/mL) was vented with a needle and 1.0–1.5 mL of the 1 M acetate buffer (pH 5.0) was added. The total volume was withdrawn using a sterile syringe and was afterwards employed for the reconstitution of the DOTA-HYNIC-iPSMA lyophilized kit. The reconstituted vial was heated in a dry bath at 95°C for 30 min. After cooling to room temperature, the vial was vented with a needle and the volume was taken up to 10 mL with 0.9% saline solution (Pisa, Mexico), using a sterile syringe. The dosing step was carried out directly in delivery syringes using a dosing GMP module (Musa, Comecer, Italy). In this way, ^177^Lu-iPSMA was obtained from lyophilized formulations after reconstitution with sterile solutions of ^177^LuCl_3_ without the need to perform further purification or sterilization processes and without the need of using commercially available radiochemical synthesizers. For the quality control, parameters such as color, appearance, pH, sterility, bacterial endotoxins, and radiochemical purity (reversed-phase HPLC) were evaluated in accordance with the Mexican Pharmacopoeia [11].

^68^Ga was obtained from a ^68^Ge/^68^Ga generator (Isotope Technologies, Garching) and Glu-CO-Lys(Ahx)-HBED-CC (PSMA-11, GMP) from ABX advanced biomedical compounds. The synthesis of ^68^Ga-PSMA-11 or ^68^Ga-iPSMA(^68^Ga-DOTA-HYNIC-iPSMA) was carried out on an iQS Ga-68 Fluidic Labeling Module (Isotope Technologies, Garching). The [^99m^Tc]Tc-pertechnetate was obtained from a GETEC ^99^Mo/^99m^Tc generator (ININ-Mexico). [^99m^Tc]Tc-EDDA/HYNIC-iPSMA ([^99m^Tc]Tc-ethylenediamine-*N*,*N*′-diacetic acid (EDDA)/hydrazinonicotinyl(HYNIC)-Lys(Nal)-Urea-Glu) was prepared from a lyophilized formulation (ININ-Mexico) as previously reported [8].

### 2.3. Evaluation of Radiochemical Purity

Radiochemical purity analyses were performed by reversed-phase high-performance liquid chromatography (HPLC) with a Waters instrument running Empower software with both radioactivity and UV-photodiode array in-line detectors and a *µ*Bondapak C_18_ column (5 *µ*m, 3.9 × 300 mm). A gradient using 0.1% TFA/water as solvent A and 0.1% TFA/acetonitrile as solvent B was used at a flow rate of 1 mL/min. The gradient began at 100% solvent A for 3 min, changed to 50% solvent A over 10 min and was maintained for 10 min, changed to 30% solvent A over 3 min and finally returned to 100% solvent A over 4 min. In this system, retention times for free ^177^LuCl_3_ and ^177^Lu-iPSMA were 3–4 min and 14–15 min, respectively. The same system was used for [^99m^Tc]Tc-iPSMA (*t*_R_ = 13–14 min; *t*_R_ = 3–4 min for [^99m^Tc]TcO_4_Na) and ^68^Ga-PSMA-11/^68^Ga-iPSMA (*t*_R_ = 10–12 min; *t*_R_ = 3-4 min for ^68^GaCl_3_) radiochemical purities (RPs) assessment in order to verify RP over 95%.

### 2.4. Healthy Subjects and Patients

Five healthy subjects (mean age ±SD, 47 ± 7 y; age range, 36–53 y; 5 men) were included. Prescreening consisted of a detailed review of medical history and a physical examination. Subjects with evidence of clinical disease or a history of organ-removal surgery were excluded. The mean (±SD) subject weight was 74 ± 8 kg (range, 64–82 kg). All subjects signed a consent form after receiving detailed information regarding the aims of the study and agreed to the collection of data that is necessary for a complete biokinetic study. The activity administered to healthy subjects was 185 MBq (from 3 to 5 *µ*g of DOTA-HYNIC-iPSMA peptide).

Ten patients (median age: 68 y; range 58–86 y) with histologically confirmed prostate cancer were enrolled in this study and received from 1 to 4 cycles of ^177^Lu-iPSMA (3.7 or 7.4 GBq, from 60 to 120 *µ*g of DOTA-HYNIC-iPSMA peptide) every 8–10 weeks. The activity to be administered in each treatment was established according to the tumor volume estimated by the SPECT/CT or PET/CT diagnostic study. For example, patients with greater bone tumor load were initially treated with 3.7 GBq due to the higher probability of myelotoxicity, with gradual scaling. The criterion to determine the number of cycles to be administered was the biochemical and imaging progression. In Tables 1 and 2, the patient characteristics before ^177^Lu-iPSMA treatments and the radiotracer used in each patient for evaluation of the therapy effect are shown. Response was evaluated using the ^68^Ga-PSMA-11 PET/CT or ^68^Ga-iPSMA PET/CT or [^99m^Tc]Tc-iPSMA-SPECT/CT diagnostic images and serum PSA levels before and after ^177^Lu-iPSMA treatment. Renal scintigraphy using [^99m^Tc]Tc-MAG3 was performed in all patients. Additional renal laboratory parameters and blood counts were performed to rule out clinically relevant impairment of renal or hepatic function and bone marrow depression. Written informed consent was obtained from each patient. Immediately after radiopharmaceutical administration, healthy subjects and patients were hydrated with 500 mL of pure water and they voided the bladder before the image acquisition. The study was approved by the hospital's Medical Ethics Committee, taking into account the following aspects: (a) the ethical standards of the responsible committee on human experimentation (institutional and national) and with the Helsinki Declaration of 1975, as revised in 2008, (b) the GMP certificate issued by COFEPRIS (Federal Commission for Protection against Health Risks, the regulatory authority in Mexico) to ININ, (c) the clinical background of PSMA inhibitors for imaging and therapy, (d) the techno-surveillance report of the [^99m^Tc]Tc-EDDA/HYNIC-iPSMA and ^177^Lu-DOTA-peptides that ININ distributes with the approval of COFEPRIS for clinical use, (e) the complete preclinical studies of ^177^Lu-iPSMA and (f) the basis of microdosing studies.

### 2.5. Acquisition of Images

^177^Lu-iPSMA images were obtained to calculate the biokinetic and dosimetry parameters with a dual head gamma camera (Symbia TruePoint SPECT/CT, Siemens), equipped with medium energy general purpose collimators. The scan velocity was 12 cm/min. For all acquisitions, a matrix size of 256 × 1024 pixels was used and a symmetric 15% window was set at 208 keV. For scatter corrections, the dual-energy window method was used by simultaneous acquisition in a lower scatter window centered on 176 keV with 15% width [13]. Transmission factors for the chest and abdomen were calculated using the ratio of the count rates *I*/*I*_0_ obtained with a 37 MBq ^177^Lu-filled flood source with (*I*) and without (*I*_0_) the patient in position, from which the regional attenuation of the body was calculated. In healthy subjects, whole-body anterior and posterior scintigraphy was performed at 20 min, 6, 24, 48, and 120 h after radiopharmaceutical administration.

In patients, whole-body planar scintigraphy ^177^Lu-iPSMA images (anterior and posterior) were obtained at 24 h after radiopharmaceutical administration. ^68^Ga-PSMA-11-PET/CT or ^68^Ga-iPSMA-PET/CT images before (basal) and after ^177^Lu-iPSMA therapy (40–50 d after therapy) were acquired in four patients and two patients, respectively. Studies were performed on an mCT Excel 20 PET/CT scanner (Siemens Medical Solutions). The acquisition parameters of the helical CT scan were 120 kVp, 180 mAs, and 5 mm slice thickness. After intravenous injection of ^68^Ga-PSMA-11 or ^68^Ga-iPSMA, whole-body emission scans were acquired at 60 min. The whole-body PET scans were obtained from the vertex to mid thighs, at 2–3 min per bed position in the 3-dimensional mode. PET images were reconstructed using a 2-dimensional ordered-subset expectation maximization algorithm. ROIs were drawn, and the maximum standardized uptake values (SUV_max_) were calculated. [^99m^Tc]Tc-EDDA/HYNIC-iPSMA SPECT/CT images before and after ^177^Lu-iPSMA treatment (40–50 d after therapy) were obtained from four patients at 3 h posttracer injection. A 360-degree rotation with a noncircular orbit continuous technique, 128 × 128 matrix, window of 15% centered on 140 keV with scattering correction, 120 images of 10 seconds in all acquisitions, were used. CT images were acquired from the skull to the middle third of thighs, obtaining an attenuation correction map using low-dose CT parameters. Reconstruction of raw data was carried out using the iterative method by the order of sets and subsets (8 iterations/4 subsets) and a Butterworth filter (0.5 cut, order 5). ROIs were drawn, and the SUV_max_ were calculated.

### 2.6. ^177^Lu-iPSMA Biokinetics

For scattering correction, the images obtained by the dual-energy window method were archived in DICOM (Digital imaging and Communication in Medicine) format and processed with the ImageJ Software (Image Processing and Analysis in Java, Version 1.51i). ROIs were drawn around source organs (heart, breast, lungs, kidneys, liver, spleen, intestine, bladder, salivary glands, lacrimal glands, and whole body) in each time frame. The same set of ROIs was used for all scans, and the counts in each ROI were corrected by attenuation using the transmission factors (*I*/*I*_0_) experimentally calculated as described above, in agreement with the conjugate-view counting method for additional scattering correction as follows [9, 13]:

(1) $$\begin{array}{c} A=\frac{I}{I_{0}}\sqrt{I_{\text{A}}}I_{\text{P}}, \end{array}$$

where *A* represents the compartment activity in counts and *I*_A_/*I*_P_ are the anterior/posterior view counting rates, respectively. Counts were also corrected by physical decay and by the background correction factor, in accordance with the Buijs method [14].

Each organ activity was divided by the whole-body (WB) activity obtained from the first image acquired (100% of injected activity), in order to determine the fraction of the injected activity (IA) in each source organ as follows:

(2) $$\begin{array}{c} \%{\text{IA}}_{\text{source organ}}=\frac{A_{\text{source organ}}}{A_{\text{WB at the first image acquisition}}}\times 100. \end{array}$$

The image sequence was used to derive ^177^Lu time-activity curves in each organ. As the heart does not overexpress PSMA, its activity was considered as having blood activity kinetics. The blood activity curve was derived from the heart activity by fitting the heart data to a function with three exponential terms. The OLINDA/EXM code allows the user to enter kinetic data for each source organ (% IA at different times) and fit it to one or more exponential terms [15]. The activity was integrated over time to calculate the total number of disintegrations (*N*) expressed per unit of initial activity in the source region (MBq^.^h/MBq). The ICRP 30 GI tract model included in the OLINDA/EXM code was used for the excretion model, assuming an activity fraction of 0.034–0.080 entering the small intestine, as images revealed that 5.9 ± 2.9% of the total activity was excreted into the intestine at 20 min after injection. The estimated bladder activity (% IA in urine) was input data for the OLINDA/EXM code. Total urine excretion at 24 h was estimated from the acquired images as follows:

(3) $$\begin{array}{c} \text{urine excretion}=\left(\frac{A_{\text{WB at }24\text{ h, corrected by decay}}}{A_{\text{WB at the first image acquisition}}}\times 100\right)-\left(\%\text{hepatobiliary excretion}\right). \end{array}$$

### 2.7. ^177^Lu-iPSMA-Absorbed Dose Calculations

The absorbed dose to organs was evaluated according to the following equation as previously reported [9]:

(4) $$\begin{array}{c} {\text{D}}_{\text{target}⟵\text{source}}=\sum_{\text{sources}}{\text{N}}_{\text{source}}\times {\text{DF}}_{\text{target}⟵\text{source}}, \end{array}$$

where D_target⟵source_ is the mean absorbed dose to a target organ from a source region, N_source_ represents the total number of nuclear transitions that occurred in the source region, and DF_target←source_ is a dose factor that is specific for the isotope, source, and target configuration. In this study, the equivalent absorbed dose estimates were obtained by entering the experimental *N* values for all source organs into the OLINDA/EXM code [15], but the effective doses were calculated according to the ICRP 103, in which salivary and lacrimal glands are included. The mass and DF-values of the salivary and lacrimal glands were obtained according to Liu et al. [16].

## 3. Results and Discussion

The radiochemical purity of ^177^Lu-iPSMA (Figure 1) obtained from multidose lyophilized kits was 99 ± 1%, as obtained by HPLC analyses without postlabeling purification. The average molar activity was 70 GBq/*µ*mol before injection to patients.

**Figure 2:**
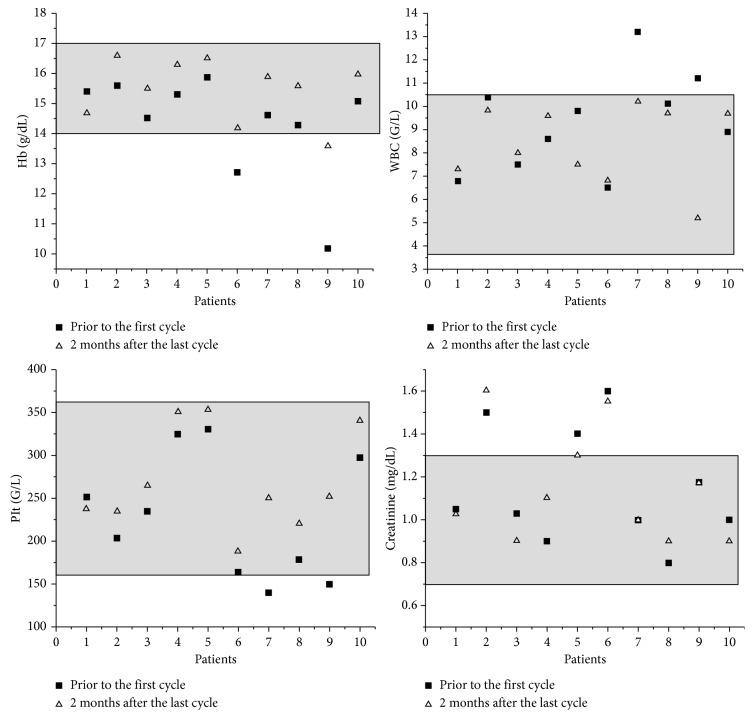
Hemoglobin levels, blood cell count, and creatinine values of patients before and after ^177^Lu-iPSMA therapy.

None of the five healthy subjects reported adverse reactions such as nausea, vomiting, dyspnea, bronchospasm, decreased blood pressure, itching, flushing, hives, chills, coughing, bradycardia, muscle cramps, or dizziness after the radiopharmaceutical was administered. No significant change in hemoglobin and the blood cell count was observed after therapy in patients, and there was no evidence of nephrotoxicity (Figure 2). Patients 2, 5, and 6 had abnormal creatinine values before the treatment which did not change after therapy. The same behavior was observed with the GFR values, which were in the normal range (72–89 mL/min) before and after treatments except in patients with renal complications (patient 2, 5, and 6, GFR range of 58–62 mL/min). Three patients (30%) showed mild reversible xerostomia following treatment. Fatigue was a side effect in two patients (20%), and nausea was observed in one man treated with a ^177^Lu-iPSMA therapeutic dose (10%) at 24 h. One patient reported complete pain relief (patient 6).

**Figure 3:**
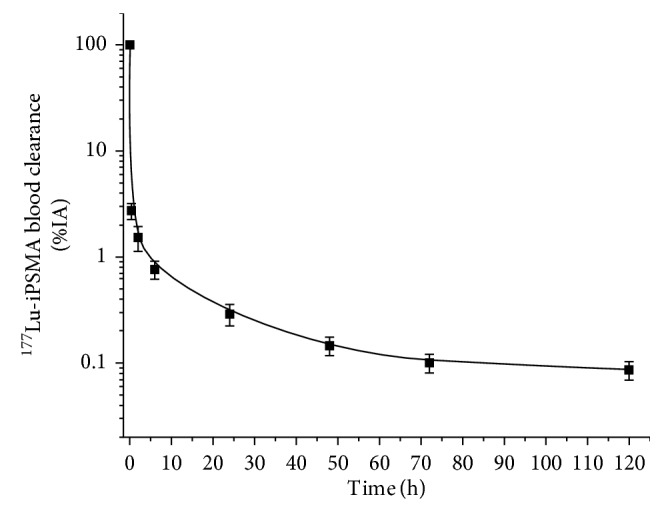
^177^Lu-iPSMA clearance from blood of healthy volunteers. The curve shows three different slopes with *T*_*1/2*_*α* = 1.1 h, *T*_*1/2*_*β* = 9.2 h, and *T*_*1/2*_*γ* = 79.6 h.

The ^177^Lu-iPSMA blood activity was fitted to a triexponential function as follows as follows (Figure 3):

(5) $$\begin{array}{c} A\left(t\right)=2.11e^{0.614\text{t}}+0.88e^{0.075\text{t}}+0.13e^{0.0081\text{t}}. \end{array}$$

**Figure 4:**
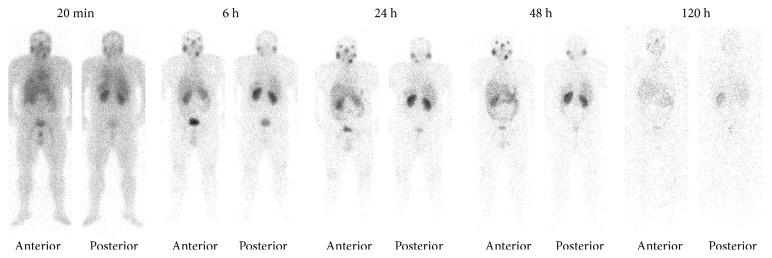
Anterior and posterior whole-body images of a healthy volunteer (man) at 20 min, 6, 24, 48, and 120 h after ^177^Lu-iPSMA administration (185 MBq).

The half-life value was 1.1  h for the fast component (*T*_1/2_*α* = ln2/0.614), 9.2 h for the first slow component (*T*_1/2_*β* = ln2/0.075), and 79.6 h for the second slow component (*T*_1/2_*γ* = ln2/0.008) (Figure 2). The activity was mainly accumulated in the kidneys, liver, and parathyroid, salivary, and lacrimal glands (Figure 4). Twenty minutes after radiopharmaceutical administration, the mean percentage of the injected activity in the kidneys was 17.58 ± 7.13% and after 24 h, it decreased to 7.01 ± 3.36%. Twenty-four hours after the administration of ^177^Lu-iPSMA, the total activity excreted in urine was 63.99 ± 10.58%.

The radiation-absorbed doses for the main source organs are shown in Table 3. ^177^Lu-iPSMA showed to have similar pharmacokinetics, dosimetry, and therapeutic response compared to other ^177^Lu-PSMA inhibitors previously informed [17, 18]. The mean radiation-absorbed doses of ^177^Lu-iPSMA in the kidney (0.88 Gy/GBq) and liver (0.28 Gy/GBq) were slightly different from that reported for [^177^Lu]Lu-PSMA-I&T (kidney = 0.75 Gy/GBq, liver = 0.12 Gy/GBq) and quite comparable with those of [^177^Lu]Lu-PSMA-617 (kidney = 0.82 Gy/GBq, liver = 0.13 Gy/GBq) [17, 18]. Patient no. 1, who received 4 cycles of ^177^Lu-iPSMA with a total activity of 18.5 GBq (Table 4), had the highest mean radiation dose of 16.28 Gy to the kidney, which is safe considering that the maximum tolerated dose or dose limit is 28 Gy (50% probability of developing severe late kidney damage within 5 y) [19]. Absorbed doses for lacrimal and salivary glands in patient 1 were 21.6 and 24.4 Gy, respectively, which have been stated as well tolerated by patients. For example, using external-beam radiation therapy, doses to glands below 26 Gy were reported safe [20].

**Figure 5:**
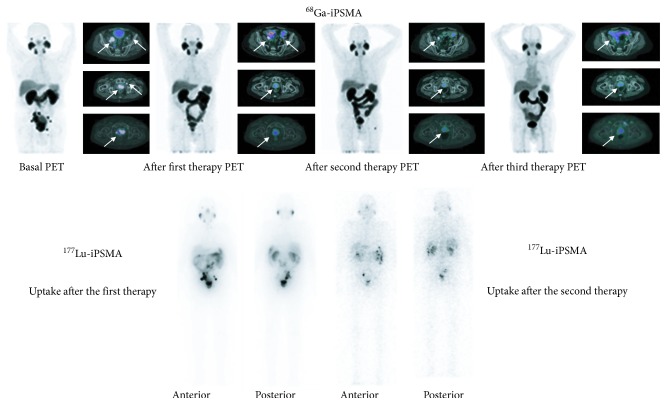
Treatment response evaluated using the ^68^Ga-iPSMA-PET/CT diagnostic images (upper images) after three cycles with ^177^Lu-iPSMA (bottom: images at 24 h after the first cycle and 6 d after the second cycle with ^177^Lu-iPSMA) (patient 1, complete response).

**Figure 6:**
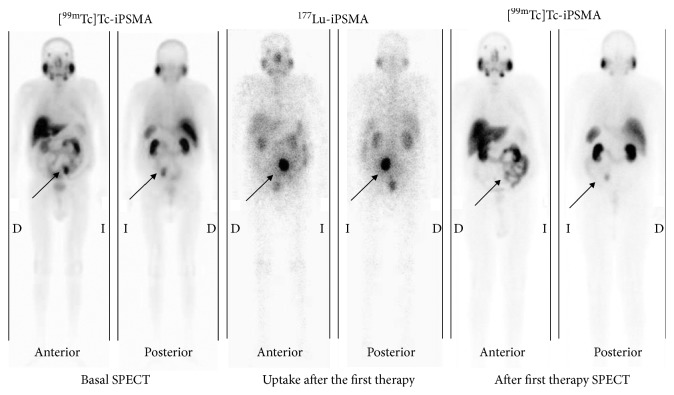
Treatment response evaluated using the [^99m^Tc]Tc-iPSMA-SPECT diagnostic images (right and left, anterior and posterior images) after the therapy with ^177^Lu-iPSMA (middle: anterior and posterior images at 24 h after the first cycle with ^177^Lu-iPSMA) (patient 4, partial response).

All patients showed high ^177^Lu-iPSMA uptake in the prostate cancer metastases (Figures 5 and 6) with an average tumor/background ratio of 6.3 ± 1.7 at 24 h. ^177^Lu-iPSMA was able to target both soft tissue tumors and bone metastatic lesions of the prostate cancer (Figure 6). A total of 18 cycles were performed in 10 patients. A PSA decrease and some reduction of the radiotracer uptake in tumor lesions (SUV_max_) occurred in 60% and 70% of the patients, respectively (4/10 patients with a PSA decrease ≥50% and 2/10 with a PSA decrease ≤42%) (Table 4). Baum et al. [4] found that 80% of all men (*n* = 56) treated with [^177^Lu]Lu-PSMA-I&T had a PSA decrease. Therefore, differences with our preliminary study could be related with the total number of enrolled patients and the total cycles of ^177^Lu-iPSMA therapies. Furthermore, Rahbar et al. [21] reported that a relevant number of patients treated with [^177^Lu]Lu-PSMA-617 showed a delayed response, even if they did not respond to the first cycle of the therapy.

**Figure 7:**
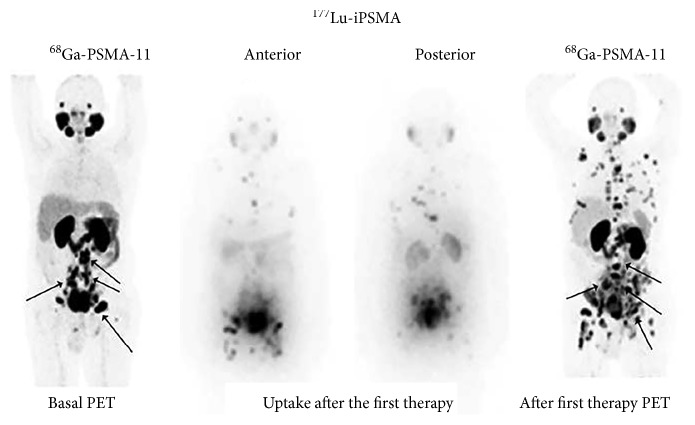
Treatment response evaluated using the ^68^Ga-PSMA-11-PET/CT diagnostic images (right and left anterior images) after the therapy with ^177^Lu-iPSMA (middle: anterior and posterior images at 24 h after the first cycle with ^177^Lu-iPSMA) (patient 7, partial response in soft tissue lesions but progressive disease in bone).

Three patients (30%) that showed a decrease in SUVs of soft tissue tumor lesions (visceral or lymph nodes) had a progressive disease mainly with an increase in bone metastases (Table 4, Figure 7). Bone metastases appeared to respond to treatment with ^177^Lu-iPSMA less well than visceral or lymph nodal disease in agreement with previous clinical studies [22]. This fact could be related with the inability of ^177^Lu-PSMA inhibitors to specifically target the homing process in the bone (bone microenvironment which promotes homing of a cancer cell to the bone) [23].

Decrease in PSA levels or SUV values were not related with previous therapeutic treatments. One patient (10%) had complete response (Figure 5).

Progressive disease despite ^177^Lu-iPSMA treatment occurred in 30% of patients (Table 4). As others have reported, this can be related to the different density expression of the PSMA receptor in all cells. Heterogeneity of PSMA receptor activity within the tumor population may mean that some sites will not respond to treatment with ^177^Lu-iPSMA, which will manifest as disease progression and a rising serum PSA level [22].

It is important to mention that, although the evaluation of ^68^Ga-iPSMA for diagnostic images was not the aim of this research, results also evidenced the feasibility of using the ^68^Ga-iPSMA/^177^Lu-iPSMA pair in theranostic applications and that ^68^Ga-PSMA-11 and ^68^Ga-iPSMA tracers have comparable ability to target the PSMA protein. Nevertheless, a complete clinical comparative study between ^68^Ga-PSMA-11 and ^68^Ga-iPSMA is needed before generating any conclusion.

Several ^68^Ga-PSMA inhibitors for PET/CT imaging in prostate carcinoma are being used in the clinical practice [5–7]. However, a single elution from a ^68^Ge/^68^Ga generator is only sufficient to prepare ^68^Ga-PSMA ligand for a few patients. This limits the number of studies that can be carried out in a single day. Technetium-99m can be produced with large activities from ^99^Mo/^99m^Tc generators to be used in the preparation of [^99m^Tc]Tc-PSMA inhibitors (SPECT/CT images) for a multitude of patients every day [24]. Furthermore, there are fewer PET cameras installed worldwide than SPECT systems. That is the reason why we developed a lyophilized formulation for the preparation of [^99m^Tc]Tc-EDDA/HYNIC-iPSMA, in which hydrazinonicotinamide (HYNIC) was proposed to act as a critical chemical group in the increase of the lipophilicity of the molecule for the coupling to hydrophobic sites of PSMA [10]. The new ^177^Lu-DOTA-HYNIC-iPSMA (^177^Lu-iPSMA) therapeutic radiopharmaceutical can potentially work as the theranostic pair of the [^99m^Tc]Tc-EDDA/HYNIC-iPSMA ([^99m^Tc]Tc-iPSMA) diagnostic agent previously reported [8, 9].

PSMA activity in tumor cells is measured by assessing the SUV values with ^68^Ga-PSMA-11, ^68^Ga-PSMA-617, or ^68^Ga-PSMA-I&T PET/CT before ^177^Lu-PSMA inhibitor therapies. The use of [^99m^Tc]Tc-iPSMA/^177^Lu-iPSMA theranostic pair (similar active molecular sequence) could be useful to establish cutoffs of SUVs with [^99m^Tc]Tc-iPSMA below which ^177^Lu-iPSMA therapy may not be effective.

Although ^177^Lu-iPSMA obtained from kit formulations showed high tumor uptake with good response rates in patients, further clinical studies in randomized trials are necessary for evaluating the effect of this therapeutic agent on the survival of patients.

## 4. Conclusions

This preliminary study suggests that radioligand therapy with ^177^Lu-iPSMA is safe, well-tolerated, and has a considerable effect on PSA levels in patients with advanced prostate cancer. Further studies are needed to evaluate response and toxicity after several therapy cycles and to determine the optimal number of cycles, as well as to assess the effect of this therapeutic agent on the survival of patients.

## Figures and Tables

**Table 1:** Patient characteristics before ^177^Lu-iPSMA treatments.

Site of metastases	Patients (18 treatments)	
	*N*	%
Bone	6	60
Lymph nodes	10	100
Liver	4	40
Lung	1	10
*Prior therapies*		
Radical prostectomy	8	80
Radiation therapy (prostate region)	8	80
Docetaxel	7	70
Cabazitaxel, abiraterone, and/or enzalutamide	7	70
Radium-223	1	10
Radiation therapy to bone	2	20

**Table 2:** Description of the reported disease of patients and the radiotracer used in each case for evaluation of the response to ^177^Lu-iPSMA therapy.

Patient no.	Age	Reported disease	Radiotracer
1	86	Prostate cancer, resistant to castration, with retroperitoneal, external, and common iliac bilateral chains lymph node metastases	^68^Ga-iPSMA
2	72	Prostate cancer, resistant to castration, with bone, lungs, liver, and lymph node metastases; right nephrectomy and adrenalectomy with resection of the diaphragm by tumor infiltration	[^99m^Tc]Tc-iPSMA
3	71	Prostate cancer, resistant to castration, with bone and lymph node metastases	[^99m^Tc]Tc-iPSMA
4	61	Prostate cancer with lymph node metastasis	[^99m^Tc]Tc-iPSMA
5	60	Prostate cancer, resistant to castration, diabetes mellitus type 2, chronic kidney disease, kidney transplant, with liver, retroperitoneal, and left iliac chain lymph nodes and vertebral T6 metastases	[^99m^Tc]Tc-iPSMA
6	72	Prostate cancer, resistant to castration, with inguinal lymph nodes and bone metastases; kidney cancer with left nephrectomy; gastric cancer	^68^Ga-PSMA-11
7	58	Prostate cancer, resistant to castration, with right sacrum bone metastasis; retroperitoneal, iliac bilateral chains, inguinal, and gluteus lymph node metastases, bladder and liver metastases (Ra-223 treatment)	^68^Ga-PSMA-11
8	65	Prostate cancer with liver and lymph node metastases	^68^Ga-PSMA-11
9	66	Prostate cancer, resistant to castration, with multiple bone metastases (radiotherapy treatment)	^68^Ga-PSMA-11
10	74	Prostate cancer, resistant to castration, with multiple lymph node metastases (brachytherapy treatment)	^68^Ga-iPSMA

**Table 3:** Average total number of disintegrations (*N*) in source organs, organ-absorbed doses, and effective dose of ^177^Lu-iPSMA, calculated from five healthy subjects (men).

Target organ	*N* (mean ± SD)	Organ doses (Gy/GBq)	
	(MBq·h/MBq)	Average	SD
Adrenals	—	0.030	0.007
Brain	—	0.024	0.005
Breasts	—	0.024	0.004
Gallbladder wall	—	0.032	0.006
LLI wall	—	0.041	0.011
Small intestine	0.300 ± 0.018	0.027	0.006
Stomach wall	—	0.027	0.006
ULI wall	—	0.044	0.010
Heart wall	—	0.049	0.005
Kidneys	2.953 ± 0.475	0.880	0.040
Liver	6.322 ± 0.352	0.280	0.090
Lungs	0.400 ± 0.098	0.030	0.010
Muscle	—	0.025	0.006
Ovaries	—	0.040	0.010
Pancreas	—	0.030	0.007
Red marrow	—	0.030	0.010
Osteogenic cells	—	0.077	0.017
Skin	—	0.024	0.004
Spleen	0.450 ± 0.036	0.232	0.070
Testes	—	0.025	0.006
Thymus	—	0.025	0.004
Thyroid	—	0.024	0.007
Salivary glands	0.225 ± 0.005	1.170	0.310
Lacrimal glands	0.041 ± 0.013	1.321	0.091
Urinary bladder wall	—	0.249	0.103
Urinary bladder	1.369 ± 0.441	—	—
Uterus	—	0.028	0.007
Remainder of the body	19.380 ± 1.550	—	—
Total body	—	0.040	0.021

**Table 4:** PSA levels in patients before and after treatment.

Patient no. (no. of cycles, total activity administered (GBq))	PSA levels (ng/mL) (SUV_max_ in soft tissue tumor lesions)	
	Before treatment	After treatment
1 (4, 18.5)	58 (73)	4 (1)
2 (2, 7.4)	53 (32)	55 (14)
3 (2, 7.4)	20 (33)	30 (17)
4 (1, 5.5)	31 (46)	18 (4)
5 (1, 7.4)	101 (38)	45 (16)
6 (2, 7.4)	46 (33)	25 (19)^*∗∗*^
7 (2, 7.4)	217 (56)	322 (21)^*∗∗*^
8 (2, 7.4)	34 (47)	11 (26)
9 (1, 7.4)	37 (101)	40 (33)^*∗∗*^
10 (1, 3.7)	182 (79)	76 (28)

^*∗∗*^Progressive disease: bone metastases.

## Data Availability

The data used to support the findings of this study are included within the article.

## References

[1] Rajasekaran A. K., Anilkumar G., Christiansen J. J. (2005). Is prostate-specific membrane antigen a multifunctional protein?. *American Journal of Physiology-Cell Physiology*.

[2] Rahbar K., Schmidt M., Heinzel A. (2016). Response and tolerability of a single dose of ^177^Lu-PSMA-617 in patients with metastatic castration-resistant prostate cancer: a multicenter retrospective analysis. *Journal of Nuclear Medicine*.

[3] Rahbar K., Ahmadzadehfar H., Kratochwil C. (2017). German multicenter study investigating ^177^Lu-PSMA-617 radioligand therapy in advanced prostate cancer patients. *Journal of Nuclear Medicine*.

[4] Baum R. P., Kulkarni H. R., Schuchardt C. (2016). 177Lu-labeled prostate-specific membrane antigen radioligand therapy of metastatic castration-resistant prostate cancer: safety and efficacy. *Journal of Nuclear Medicine*.

[5] Eder M., Neels O., Müller M. (2014). Novel preclinical and radiopharmaceutical aspects of [68Ga] Ga-PSMA-HBED-CC: a new PET tracer for imaging of prostate cancer. *Pharmaceuticals*.

[6] Weineisen M., Schottelius M., Simecek J. (2015). 68Ga-and 177Lu-labeled PSMA I and T: optimization of a PSMA-targeted theranostic concept and first proof-of-concept human studies. *Journal of Nuclear Medicine*.

[7] Afshar-Oromieh A., Haberkorn U., Schlemmer H. (2014). Comparison of PET/CT and PET/MRI hybrid systems using a 68Ga-labelled PSMA ligand for the diagnosis of recurrent prostate cancer: initial experience. *European Journal of Nuclear Medicine and Molecular Imaging*.

[8] Ferro-Flores G., Luna-Gutiérrez M., Ocampo-Garcia B. (2017). Clinical translation of a PSMA inhibitor for ^99m^Tc-based SPECT. *Nuclear Medicine and Biology*.

[9] Santos-Cuevas C., Davanzo J., Ferro-Flores G. (2017). ^99m^Tc-labeled PSMA inhibitor: biokinetics and radiation dosimetry in healthy subjects and imaging of prostate cancer tumors in patients. *Nuclear Medicine and Biology*.

[10] Ferro-Flores G., Ocampo-Garcia B., Santos-Cuevas C. ^99m^Tc-EDDA/HYNIC-iPSMA as a radiopharmaceutical for detecting the overexpression of prostate-specific membrane antigen.

[11] Luna-Gutierrez M., Jimenez-Hernandez T., Serrano-Espinoza L. (2017). Freeze-dried multi-dose kits for the fast preparation of ^177^Lu-Tyr^3^-octreotide and ^177^Lu-PSMA(inhibitor) under GMP conditions. *Journal of Radioanalytical and Nuclear Chemistry*.

[12] Jimenez-Hernandez T., Ferro-Flores G., Ocampo-Garcia B. (2018). ^177^Lu-DOTA-HYNIC-Lys(Nal)-Urea-Glu: synthesis and in vitro and in vivo assessment to target the prostate-specific membrane antigen. *Journal of Radioanalytical and Nuclear Chemistry*.

[13] Siegel J. A., Thomas S. R., Stubbs J. B. (1999). MIRD pamphlet no. 16: techniques for quantitative radiopharmaceutical biodistribution data acquisition and analysis for use in human radiation dose estimates. *Journal of Nuclear Medicine*.

[14] Buijs W. C., Siegel J. A., Boerman O. C. (1998). Absolute organ activity estimated by five different methods of background correction. *Journal of Nuclear Medicine*.

[15] Stabin M. G., Sparks R. B., Crowe E. (2005). OLINDA/EXM: the second-generation personal computer software for internal dose assessment in nuclear medicine. *Journal of Nuclear Medicine*.

[16] Liu B., Huang R., Kuang A. (2011). Iodine kinetics and dosimetry in the salivary glands during repeated courses of radioiodine therapy for thyroid cancer. *Medical Physics*.

[17] Afshar-Oromieh A., Hetzheim H., Kratochwil C. (2015). The theranostic PSMA ligand PSMA-617 in the diagnosis of prostate cancer by PET/CT: biodistribution in humans, radiation dosimetry, and first evaluation of tumor lesions. *Journal of Nuclear Medicine*.

[18] Okamoto S., Thieme A., Allmann J. (2017). Radiation dosimetry for 177Lu-PSMA-I and T in metastatic castration-resistant prostate cancer: absorbed dose in normal organs and tumor lesions. *Journal of Nuclear Medicine*.

[19] Emami B., Lyman J., Brown A. (1991). Three-dimensional photon treatment planning report of the collaborative working group on the evaluation of treatment planning for external photon beam radiotherapy tolerance of normal tissue to therapeutic irradiation. *International Journal of Radiation Oncology∗Biology∗Physics*.

[20] Hey J., Setz J., Gerlach R. (2011). Parotid gland-recovery after radiotherapy in the head and neck region: 36 months follow-up of a prospective clinical study. *Radiation Oncology*.

[21] Rahbar K., Bogeman M., Yordanova A. (2018). Delayed response after repeated 177Lu-PSMA-617 radioligand therapy in patients with metastatic castration resistant prostate cancer. *European Journal of Nuclear Medicine and Molecular Imaging*.

[22] Emmett L., Willowson K., Violet J. (2017). Lutetium177 PSMA radionuclide therapy for men with prostate cancer: a review of the current literature and discussion of practical aspects of therapy. *Journal of Medical Radiation Sciences*.

[23] Mishra A., Shiozawa Y., Pienta K. J. (2011). Homing of cancer cells to the bone. *Cancer Microenvironment*.

[24] Lawa I. O., Ankrah A. O., Mokgoro N. P. (2017). Diagnostic sensitivity of Tc-99m HYNIC-PSMA SPECT/CT in prostate carcinoma: a comparative analysis with Ga-68 PSMA PET/CT. *Prostate*.

